# Using Influenza Vaccination Location Data from the 2018 Behavioral Risk Factor Surveillance System (BRFSS) to Expand COVID-19 Vaccination Coverage

**DOI:** 10.3390/ijerph18157753

**Published:** 2021-07-22

**Authors:** Victoria Fonzi, Kiran Thapa, Kishor Luitel, Heather Padilla, Curt Harris, M. Mahmud Khan, Glen Nowak, Janani Rajbhandari-Thapa

**Affiliations:** 1 Department of Health Policy and Management, College of Public Health, University of Georgia, Athens, GA 30602, USA; Victoria.Fonzi@uga.edu (V.F.); Mahmud.Khan@uga.edu (M.M.K.); 2 Department of Epidemiology and Biostatistics, College of Public Health, University of Georgia, Athens, GA 30602, USA; Kiran.Thapa@uga.edu; 3 School of Agriculture, Middle Tennessee State University, Murfreesboro, TN 37132, USA; kishor.luitel@mtsu.edu; 4 Department of Health Promotion & Behavior, College of Public Health, University of Georgia, Athens, GA 30602, USA; hmpadilla@uga.edu; 5 Institute for Disaster Management, College of Public Health, University of Georgia, Athens, GA 30602, USA; cuharris@uga.edu; 6 Grady College Center for Health and Risk Communication, Grady College of Journalism and Mass Communication, University of Georgia, Athens, GA 30602, USA; gnowak@uga.edu

**Keywords:** COVID-19 vaccine, vaccine distribution, vaccination location, doctor’s office, store

## Abstract

Effective COVID-19 vaccine distribution requires prioritizing locations that are accessible to high-risk target populations. However, little is known about the vaccination location preferences of individuals with underlying chronic conditions. Using data from the 2018 Behavioral Risk Factor Surveillance System (BRFSS), we grouped 162,744 respondents into high-risk and low-risk groups for COVID-19 and analyzed the odds of previous influenza vaccination at doctor’s offices, health departments, community settings, stores, or hospitals. Individuals at high risk for severe COVID-19 were more likely to be vaccinated in doctor’s offices and stores and less likely to be vaccinated in community settings.

## 1. Introduction

Recent COVID-19 response efforts have focused on improving COVID-19 vaccine distribution and administration. As of June 2021, 54% of Americans have received at least one dose of a COVID-19 vaccine, and 46% have been fully vaccinated [1]. Of the vaccines administered in the United States (US), over 95% have been mRNA-based vaccines developed by Pfizer-BioNTech or Moderna [1]. The COVID-19 vaccine from Pfizer is approved by the Federal Drug Administration (FDA) for individuals aged 12 years and older [2], whereas the Moderna COVID-19 vaccine is approved for individuals 18 years of age and older [3]. The development and distribution of these vaccines have been shaped by the urgency of the pandemic, which has prompted the US government to allocate billions in funding for vaccine research, development, distribution, and education [4]. COVID-19 vaccination efforts have relied heavily on pandemic-specific innovations, such as beginning vaccine manufacturing simultaneously with clinical trials, expanding healthcare services in varying clinical practices, and establishing mass vaccination centers [5,6].

As the number of people vaccinated continues to increase, the supply of the COVID-19 vaccine is expected to outpace demand in the US [7]. Once this occurs, the landscape of COVID-19 vaccination efforts will likely change significantly. Individuals who remain unvaccinated will primarily be vaccine-hesitant or who did not initially receive the vaccine due to poor access. Therefore, expanding vaccination coverage beyond initial efforts will require more concerted strategies to reduce barriers to obtaining the vaccine and increase vaccination acceptance. Additionally, the emergence of new SARS-CoV-2 variants may necessitate COVID-19 booster vaccines [8]. Moderna and Pfizer-BioNTech have already developed third dose vaccines against new variants, and these booster vaccines are currently undergoing clinical trials [9,10]. If these vaccines are approved, their distribution may be more similar to that of other seasonal vaccines, such as the influenza shot [11]. In developing a COVID-19 vaccination strategy to fit this evolving landscape, previous influenza vaccination efforts may be a helpful guide in informing current planning to reach key patient populations.

Influenza immunization campaigns in the US have a long history from which to draw upon, dating back to the 1940s [12]. Influenza vaccines have effectively reduced disease burden, saving an estimated 60,000 lives over the last decade alone [13]. Currently, the influenza vaccine is available at a wide variety of locations, most commonly doctor’s offices, pharmacies, stores, and workplaces [14,15]. Lu et al. have shown that patients tend to receive their influenza vaccine at the same location every year [15]. Therefore, examining the locations in which patients have previously received vaccinations may help to determine locations in which patients are familiar with receiving vaccines and have felt comfortable going for vaccines in the past. This familiarity may help encourage patients who were previously vaccine-hesitant to take the COVID-19 vaccine [16]. In addition, locations highly utilized by target populations in the past are likely the most accessible locations for these patients to receive the COVID-19 vaccine and future boosters.

Current literature on preferred vaccination location has examined populations by demographic characteristics. The doctor’s office is the most commonplace for adults to receive an influenza vaccine [14,15,17]. Nonmedical settings are also important, with pharmacies/stores and workplaces serving as the second and third most common locations, respectively, for vaccination [14,15]. Location preferences vary by racial/ethnic group, with most Hispanic and non-Hispanic black Americans vaccinated in medical settings and most non-Hispanic white Americans vaccinated in nonmedical settings [15]. People over the age of 65 also prefer medical settings [14,15].

Several studies have also examined predictors of influenza vaccination and ways to improve vaccination coverage. However, lesser known are the factors affecting people’s choice of location for vaccination. Employing Anderson’s Behavioral Model of Health Services Use [18], a study identified predisposing (e.g., age, gender, race, and ethnicity), enabling (e.g., education, health insurance), need (e.g., self-rated health, having chronic conditions) and environmental (e.g., policy adoption) factors that affected vaccination at pharmacy-based stores [19]. Empirical evidence suggests a key role of non-traditional vaccination locations such as store and community settings in increasing vaccine coverage among certain population groups such as those who are white, employed, living in a Metropolitan Statistical Area (MSA), or who have a higher income or higher education [20]. The differences in choice of place of vaccination among population groups may be influenced by convenience, proximity to the place of vaccination, cost and operation hours, and availability of and access to health personnel [21,22,23].

The current Center for Disease Control and Prevention (CDC) COVID-19 Vaccine Program Playbook prioritizes vaccinating those at highest risk of severe disease [24]. These populations include residents of long-term care facilities, individuals 65 years of age or older, and people with underlying medical conditions [24]. A number of chronic conditions may place a patient in this high-risk group, including heart conditions, cancer, chronic obstructive pulmonary disease (COPD), kidney disease, and Type 2 diabetes [25]. About 45% of patients at high-risk for COVID-19 fall into this category due to underlying medical conditions [26]. Many of these conditions, including heart conditions, COPD, and kidney disease, have previously been studied as risk factors that increase the potential for influenza-related complications [27]. Historically, adults with high-risk conditions have been vaccinated for influenza at a higher rate than adults without these conditions, although vaccination rates among both groups have fallen short of national goals [28]. Comprehensive data on COVID-19 vaccination rates among adults with chronic conditions are not yet available.

To date, we are not aware of a study that describes where people with underlying medical conditions prefer to receive vaccinations. This is critical if we are to effectively target future vaccinations to locations that will reach the greatest number of high-risk individuals. This study aims to fill this gap by examining the influenza vaccination location of individuals who are at risk for severe COVID-19 based on CDC definitions. This information can be used to inform strategies for expanding current COVID-19 vaccination coverage and future distribution of boosters.

## 2. Methods

### 2.1. Data and Participants

For this cross-sectional study, we used data from the Behavioral Risk Factor Surveillance System (BRFSS) 2018. It is the latest BRFSS survey that included questions about the location of vaccination as a core component. BRFSS, an annual telephone-based survey, collects self-reported information from non-institutionalized US adult populations (aged 18 years and older) on several health-related risk factors including chronic health conditions. BRFSS also collects data on influenza vaccination and influenza vaccination location during the last 12 months. In 2018, 437,436 non-institutionalized adults responded to the question on vaccination, of which 164,092 (weighted %: 33.1) reported “yes” to having received an influenza vaccination (nasal spray or injection) during the past year. Individuals who declined to answer the place of vaccination question or who received a vaccination outside of the US (*n* = 1348) were excluded from the analysis, yielding an analytic sample of 162,744 respondents (Supplementary Figure S1).

### 2.2. Measures

#### 2.2.1. Vaccination Location

Among those reporting receipt of influenza vaccination, respondents were asked “At what kind of place did you get your last flu vaccination?” with the following categories as responses: (1) doctor’s office or health maintenance organization (HMO), (2) health department, (3) another type of clinic or health center (community health center), (4) senior, recreation, or community center, (5) store (supermarket, drug store), (6) hospital (inpatient or outpatient), (7) emergency room, (8) workplace, (9) some other kind of place, (10) school. In this study, we categorized place of vaccination into five categories as: (1) doctor’s office/HMO, (2) health department/another type of clinic or health center (hereafter referred to collectively as “health department”), (3) senior, recreation, or community center/workplace/school (hereafter referred to collectively as “community setting”), (4) store, and (5) hospital/emergency room.

#### 2.2.2. Defining Population Groups at Risk of Severe Illness from COVID-19

We used age, body mass index (BMI), smoking behavior, and selected chronic health conditions to define at-risk population groups for severe COVID-19 illnesses consistent with current CDC guidelines [25]. BRFSS respondents are non-institutionalized adults 18 years or older; hence this study does not include the vaccination location of populations under 18. For this study, age was categorized into three groups: 18–49 years, 50–64 years, and 65+ years. This categorization was based on the differences in risk for COVID-19-related hospitalization and death based on age. The hospitalization and death rate increases 4-fold and 35-fold, respectively for individuals 50–64 years of age, and 6-fold and 95-fold, respectively for individuals 65–74 years of age, compared to the 18–29-year-old reference age group [29]. BMI was dichotomized into “Underweight/Normal Weight” (<25 kg/m^2^) and “Overweight/Obesity” (≥25 kg/m^2^). We combined overweight and obesity as both increase the risk for severe COVID-19 related illness [25]. Current smoking status was categorized as “Yes” and “No”. For chronic conditions, respondents were categorized as having “3+ chronic conditions”, “1 or 2 chronic conditions”, and “none”. The chronic conditions were selected based on the CDC’s list of conditions that put an adult at increased risk of severe illness from COVID-19 [25]. Chronic conditions included self-reported myocardial infarction, coronary heart disease, cancer (other than skin cancer), COPD, kidney disease, and diabetes (including gestational diabetes). Based on the four risk factors, we formed two COVID-19 risk categories: (1) high-risk, which included respondents having any combination of at least two of the four risk factors (*n =* 61,794), and (2) low-risk, which included participants with one or none of the four risk factors (*n =* 89,226). This is based on the assumption that people with two or more risk factors (e.g., older age and smoking) are at greater risk of severe illness and mortality associated with COVID-19 compared to those with a single risk factor (e.g., older age, or smoking).

### 2.3. Covariates

Several variables were identified from the variables included in the BRFSS that may affect people’s choice of the place of vaccination. In our study, race was categorized into six groups: Hispanic, non-Hispanic white, non-Hispanic black, non-Hispanic Asian, non-Hispanic multiracial and non-Hispanic other. Other covariates included sex (male/female), marital status (never married or a member of an unmarried couple/divorced, widowed, or separated/married), health insurance coverage (yes/no), metropolitan (MSA) status (in the center of an MSA/outside the center city of an MSA but inside the county containing the center city/inside a suburban county of an MSA/not in an MSA), and annual household income (less than 25,000/25,000 or more).

## 3. Analysis

Survey weights were used to account for the complex BRFSS survey design and to generate representative results. All estimates are presented as weighted estimates. Descriptive cross-tabulation of percentages and 95% confidence interval (CIs) of the place of vaccination by race and COVID-19-related risk was conducted to describe the differences in vaccination location by risk groups. Z-tests were performed to test whether there was significant difference in proportion of risk groups in regards to their place of vaccination. We then developed multivariable, multinomial logistic regression models to examine the association of COVID-19 risk with vaccination location. To do this, first, using “survey” package in R, replicate weights for the survey were created using bootstrapping. This helps estimate variance in an unbiased way for complex survey designs. Then, a multinomial model was fitted using “nnet” package and the regression coefficients were extracted and exponentiated to obtain odds ratios. The multinomial logistic regression can be illustrated using the following equation: ln ($\pi_{\mathrm{jc}}/\pi_{\mathrm{jr}}$) = $\alpha_{\mathrm{jc}}+\beta_{\mathrm{jc}}X$, where, j represents the number of outcome categories, $\pi_{\mathrm{jc}}/\pi_{\mathrm{jr}}$ represents the logit of the outcome comparing a particular outcome category to the reference category, X is a linear set of predictors, and α and β represent intercept and coefficients respectively [30]. This model allows us to assess the probability of a respondent’s choice for a particular place of vaccination (compared to the referent group) depending on the values of the independent variables. The outcomes in our regression analysis were five unordered categories representing the place of influenza vaccination. We compared the community, doctor’s office, store, and state-affiliated vaccination locations with hospitals (reference group). First, we ran the model with independent risk factors and then with COVID-19 risk groups. Observations with missing data or “don’t know” or “refused” responses in any of the study variables were dropped for the regression analysis. The regression analyses were adjusted for all the covariates listed above. Observations with missing values on each study variable are reported in Figure S1 in the supplementary file. The publicly available BRFSS survey data are anonymized and hence required no institutional review board evaluation. P values less than 0.05 were considered statistically significant. All analyses were conducted in R version 4.0.3 (http://www.R-project.org (accessed on 29 June 2021)).

## 4. Results

Table 1 shows the percentage (95% confidence interval) of flu-vaccinated populations by vaccination location for each COVID-19 risk category and for individual risk factors. Overall, 39.1% of the respondents received their influenza vaccination in a doctor’s office, followed by 26.2% in stores. The proportion of the population receiving a vaccination in a doctor’s office was higher among the high-risk group (46.4%) than the low-risk group (35.8%) (*p* < 0.001). In comparison, 31.2% of the high-risk group and 24.3% of the low-risk group were vaccinated at a store (*p* < 0.001). A larger percentage of the low-risk (20.4%), as opposed to the high-risk group (7.3%), received vaccinations in community settings (*p* < 0.001). The doctor’s office was the most common place of vaccination for all races. Among Hispanic populations, a health department was the second most common place of vaccination (20.8%); among other races, a store was the second most common vaccination location. Among those with an annual household income <25,000 USD, the health department was the third most common place of vaccination (20.3%) behind doctor’s offices and stores. Among those with an annual household income ≥25,000 USD, the third most common place of vaccination was community settings.

Table 2 shows the multinomial regression results for preferred place of vaccination by independent risk factors. Those with chronic health conditions had significantly lower odds of receiving a vaccination in a community setting (OR: 0.66, 95% CI: 0.52, 0.84 for 1–2 chronic conditions; OR: 0.35, 95% CI: 0.19, 0.63 for >2 chronic conditions) than at a hospital. Those with two or more chronic conditions also had lower odds of receiving a vaccination at a store (OR: 0.57, 95% CI: 0.34, 0.94) than at a hospital. Those who did not smoke had lower odds of vaccination at a doctor’s office (OR: 0.65, 95% CI: 0.47, 0.90) and at a store (OR: 0.69, 95% CI: 0.49, 0.96) compared to a hospital. Similarly, adults over 65 years of age (vs. 18–49 years of age) had higher odds of getting vaccinated at a doctor’s office (OR: 2.03, 95% CI: 1.42, 2.91) and a store (OR: 1.85, 95% CI: 1.28, 2.68) and lower odds of getting vaccinated in a community setting (OR: 0.35, 95% CI: 0.22, 0.55) compared to a hospital.

Table 3 shows the multinomial regression results for place of vaccination by COVID-19 risk groups. Those at high COVID-19 risk (as opposed to those at low risk) had significantly higher odds of receiving an influenza vaccination at a doctor’s office (OR: 1.52, 95% CI: 1.21, 1.91), a store (OR: 1.40, 95% CI: 1.12, 1.76), or a health department (OR: 1.37, 95% CI: 1.06, 1.77) and had significantly lower odds of receiving a vaccination in community settings (OR: 0.54, 95% CI: 0.41, 0.70) than at hospitals.

## 5. Discussion

Our results suggest that doctors’ offices are the most common locations for influenza vaccination by adults over the age of 65 and those at high risk for COVID-19. These findings are in line with previous studies that show a preference for vaccination at doctors’ offices among seniors [14], those at high risk for severe influenza infection [23], and the general population [15]. Multiple studies have found that a patient is more likely to receive an immunization after receiving a physician recommendation, evidence which may explain this consistent preference for doctors’ offices [31,32,33]. Health education by primary care physicians can also help to overcome vaccine hesitancy, as 51% of vaccine-hesitant adults trust their primary care provider to give reliable information about vaccines [34]. However, multiple factors beyond lack of information contribute to vaccine hesitancy, including a belief that the vaccine is unnecessary, a desire to take a “wait-and-see” approach, deterrence by small logistical barriers, or distrust of the vaccine development process [32]. Therefore, health education by medical providers must be combined with additional individual- and organization-level interventions such as tailored vaccine reminders, patient educational materials, and removal of barriers to vaccination to successfully overcome multiple contributors to vaccine hesitancy [35].

Stores may serve as an important secondary location for COVID-19 vaccination efforts. The US Department of Health and Human Services has authorized state-licensed pharmacists to order and administer COVID-19 vaccines [36]. In our study, individuals over the age of 65 and those at high risk for severe COVID-19 disease showed a preference for receiving influenza vaccinations at stores. A similar preference for store influenza vaccination was previously reported for seniors during the 2014–2015 influenza season [14]. Store pharmacies have a number of advantages as vaccination centers, including flexible staffing, the ability to utilize retail space if needed, and previous experience conducting influenza vaccine clinics [37]. About nine in ten Americans live within five miles of a community pharmacy, making them geographically accessible as well [38]. However, one major downside to distributing vaccines through stores is that many patients are unaware that pharmacists can give vaccines [39]. As a result, extra care would need to be taken to advertise in-store vaccination opportunities to the public.

One consistent finding across multiple COVID-19 vaccine priority groups was a lower likelihood of seeking vaccination at community centers, workplaces, and schools. Seniors over the age of 65, patients with one or more chronic conditions, and those at high risk of severe COVID-19 disease were all less likely to be vaccinated in one of these community settings compared to individuals in lower-risk groups. This result makes sense, as many retired adults who fall into these at-risk categories do not regularly visit a workplace or school, making these inefficient locations for vaccination efforts. In addition, even for the high-risk patients who frequently visit workplaces or schools, vaccine uptake may be low at these locations. An online national survey of 1007 participants found that only 45% of people who were offered an influenza vaccination at their workplace were vaccinated [40]. While this influenza vaccination rate is higher than that of the overall study population (31%), these figures indicate that fewer than half of employees who are aware of workplace influenza vaccination opportunities take advantage of them [40]. Key reasons for this vaccine hesitancy include lack of recommendation from medical personnel and fear of vaccine-adverse events [33], concerns which patients may not feel are addressed in non-clinical community settings. Therefore, if these venues are widely used for future COVID-19 vaccine administration, health communication efforts must emphasize that these non-clinical settings are as safe and trustworthy for vaccination as more familiar medical settings. This could include bringing in clinicians to answer questions and administer the vaccine, conducting a clinician led workshop for employees to gain information and ask questions, providing paid sick leave for adverse immune responses, or providing incentives for taking the vaccine.

The World Health Organization (WHO) Behavioral and Social Drivers (BeSD) Increasing Vaccination Model describes the decision to be vaccinated as a combination of motivational and practical factors [32]. Vaccine hesitancy may occur when either type of factor is absent [32]. Expanding the future distribution of vaccines to doctors’ offices and stores has the potential to address both of these concerns. A physician recommendation may act as a strong motivating factor for vaccination for patients who lack the willingness or intention to receive a COVID-19 vaccine [32,41]. It is also crucial to involve other clinical professionals such as advanced practice providers, nurses, and pharmacists and community level leaders, e.g., faith-based organization leaders to support physician recommendations. Among patients for whom practical factors are a larger barrier to vaccination, the availability of vaccine clinics in local stores may increase the convenience and accessibility of the COVID-19 vaccine. As a result, expanding existing federal partners collaborations with private sector pharmacies [42] may help to facilitate future vaccine uptake. Because patients already have a familiarity with receiving vaccinations at doctors’ offices and stores, focusing distribution efforts at these locations may help to normalize COVID-19 vaccination [43] and maximize efforts to increase vaccination coverage.

This study has several inherent limitations. First, BRFSS data are self-reported and is often collected weeks or months after influenza vaccination, so data may be subject to recall bias. However, the sensitivity of self-reported influenza vaccination has been shown to be relatively high when compared with electronic medical records, particularly among at-risk populations [44]. Second, response rates for the 2018 BRFSS were relatively low, 53% for landlines and 43% for cell phones, creating the potential for nonresponse bias. Third, healthcare workers who were vaccinated at their workplace had to choose between reporting “workplace” or “doctor’s office”/“hospital” as their place of vaccination, potentially lowering estimates for both workplace and medical setting vaccination rates. Fourth, the BRFSS does not ask for respondent rationale for choosing a particular vaccination location, so it is impossible to draw a conclusion about whether accessibility or personal preference played a larger role in deciding where to seek a vaccine [14]. Finally, the BRFSS does not collect data on some chronic conditions that would place an individual in the high-risk category, including dementia, sickle cell disorder, substance use disorder, HIV, or other immunocompromising conditions. Therefore, the number of individuals in the high-risk category may be underestimated.

## 6. Conclusions

Populations at high risk of severe COVID-19 disease were more likely to receive influenza vaccinations at doctor’s offices and stores, indicating that these locations are familiar and accessible places to receive a COVID-19 vaccine or booster for this target population. In planning for the distribution of remaining first dose COVID-19 vaccines and potential future booster vaccines, doctor’s offices and stores should be prioritized to more effectively reach high-risk individuals. We found that people at high risk of severe COVID-19 were less likely to receive previous vaccinations at community centers, making these locations less promising as future COVID-19 vaccine distribution sites. The results are generally valid because other covariates considered important in the choice of the place of vaccination have been controlled for, and the complex survey design has been taken into account to allow for more precise estimation of variance.

## Figures and Tables

**Table 1:** Place of vaccination by COVID-19 risk groups and individual risk factors among US adult population, BRFSS 2018.

Population Characteristics	Doctor’s Office/HMO (*N =* 62,830)	Health Department (*N =* 17,757)	Community Centers (*N =* 25,093)	Store (*N =* 47,234)	Hospital (*N =* 9830)
Overall	39.05 (38.53, 39.57)	10.37 (10.03, 10.71)	16.54 (16.16, 16.92)	26.19 (25.75, 26.63)	7.86 (7.54, 8.18)
COVID-19 risk					
Low risk	35.84 (35.17, 36.51)	10.93 (10.50, 11.36)	20.37 (19.84, 20.90)	24.27 (23.70, 24.83)	8.60 (8.17, 9.03)
High risk	46.40 (45.48, 47.32)	8.82 (8.23, 9.40)	7.32 (6.87, 7.78)	31.19 (30.38, 32.00)	6.27 (5.72, 6.82)
Chronic conditions					
None	35.20 (34.56, 35.84)	10.55 (10.13, 10.96)	20.54 (20.02, 21.06)	25.50 (24.94, 26.05)	8.22 (7.80, 8.64)
1–2	45.92 (44.99, 46.86)	9.90 (9.31, 10.49)	9.74 (9.21, 10.26)	27.54 (26.75, 28.32)	6.90 (6.42, 7.38)
>2	47.86 (45.19, 50.54)	10.93 (8.78, 13.07)	5.07 (4.05, 6.09)	27.01 (24.74, 29.28)	9.13 (7.05, 11.22)
Smoking					
No	38.85 (38.30, 39.40)	10.33 (9.97, 10.69)	16.74 (16.33, 17.15)	26.40 (25.93, 26.87)	7.68 (7.34, 8.01)
Yes	40.91 (39.34, 42.48)	10.71 (9.73, 11.68)	14.96 (13.88, 16.05)	24.05 (22.69, 25.42)	9.37 (8.15, 10.58)
BMI category					
Normal	37.65 (36.65, 38.64)	10.00 (9.42, 10.58)	16.34 (15.66, 17.01)	27.85 (27.00, 28.70)	8.16 (7.55, 8.78)
Above normal	39.76 (39.13, 40.39)	10.27 (9.85, 10.69)	16.56 (16.08, 17.04)	25.70 (25.16, 26.24)	7.71 (7.31, 8.10)
Age category					
18–49 years	31.45 (30.50, 32.39)	13.35 (12.66, 14.03)	24.91 (24.04, 25.77)	19.82 (19.01, 20.64)	10.48 (9.82, 11.13)
50–64 years	39.04 (38.04, 40.03)	9.15 (8.58, 9.71)	18.73 (18.01, 19.45)	24.96 (24.13, 25.79)	8.13 (7.42, 8.84)
65+ years	46.97 (46.12, 47.81)	8.34 (7.81, 8.88)	5.30 (4.96, 5.63)	34.25 (33.50, 35.01)	5.14 (4.73, 5.54)
Race					
White	39.44 (38.90, 39.97)	8.04 (7.76, 8.32)	17.18 (16.77, 17.59)	29.22 (28.74, 29.71)	6.12 (5.82, 6.42)
Black	44.34 (42.48, 46.19)	11.63 (10.32, 12.95)	15.43 (14.08, 16.79)	16.32 (15.11, 17.53)	12.28 (10.97, 13.58)
Others	35.47 (32.09, 38.85)	18.20 (16.06, 20.34)	17.16 (14.42, 19.90)	19.60 (16.90, 22.29)	9.57 (7.77, 11.37)
Asian	36.76 (33.25, 40.27)	10.83 (8.55, 13.10)	17.94 (15.40, 20.48)	20.60 (17.77, 23.43)	13.87 (11.30, 16.45)
Multiracial	36.03 (32.42, 39.63)	15.53 (12.79, 18.27)	15.74 (13.03, 18.44)	23.75 (20.28, 27.23)	8.95 (6.55, 11.35)
Hispanic	34.12 (32.21, 36.03)	20.75 (19.18, 22.33)	13.41 (12.18, 14.64)	20.69 (19.15, 22.24)	11.02 (9.80, 12.24)
Sex					
Female	40.61 (39.91, 41.30)	9.88 (9.43, 10.33)	16.23 (15.73, 16.73)	25.91 (25.32, 26.51)	7.37 (6.95, 7.79)
Male	37.12 (36.34, 37.90)	10.98 (10.47, 11.50)	16.90 (16.31, 17.49)	26.53 (25.86, 27.20)	8.47 (7.96, 8.97)
Marital status					
Never married	34.91 (33.67, 36.16)	13.22 (12.37, 14.06)	19.63 (18.61, 20.64)	22.17 (21.13, 23.21)	10.07 (9.29, 10.85)
Married	38.24 (37.56, 38.92)	10.02 (9.54, 10.49)	17.65 (17.13, 18.17)	26.87 (26.28, 27.46)	7.22 (6.81, 7.63)
Others ^a^	45.66 (43.61, 45.70)	8.74 (8.24, 9.25)	11.11 (10.51, 11.72)	28.06 (27.17, 28.95)	7.42 (6.71, 8.14)
Health plan					
No	29.25 (26.80, 31.70)	25.81 (23.19, 28.43)	15.90 (13.83, 17.96)	19.18 (17.18, 21.18)	9.86 (8.31, 11.41)
Yes	39.51 (38.98, 40.04)	9.59 (9.26, 9.91)	16.59 (16.20, 16.98)	26.56 (26.11, 27.02)	7.75 (7.42, 8.08)
MSA status					
Center city	43.21 (41.74, 44.67)	8.41 (7.49, 9.34)	11.61 (10.81, 12.42)	31.50 (30.22, 32.78)	5.27 (4.56, 5.97)
Outside ^b^	43.79 (41.97, 45.62)	6.28 (5.40, 7.16)	12.12 (10.95, 13.29)	32.34 (30.62, 34.05)	5.48 (4.39, 6.56)
Suburban ^c^	45.25 (43.22, 47.28)	6.68 (5.82, 7.55)	13.16 (11.73, 14.58)	29.53 (27.97, 31.09)	5.38 (4.21, 6.55)
Not in an MSA	43.37 (41.89, 44.85)	12.83 (11.74, 13.91)	11.93 (11.01, 12.84)	26.45 (25.28, 27.62)	5.43 (4.78, 6.07)
Income level (in USD)					
<25,000	43.06 (41.87, 44.24)	15.39 (14.47, 16.30)	8.53 (7.83, 9.24)	24.40 (23.38, 25.41)	8.62 (7.89, 9.35)
≥25,000	36.89 (36.26, 37.51)	9.06 (8.66, 9.46)	20.32 (19.82, 20.82)	25.97 (25.44, 26.50)	7.76 (7.37, 8.15)
Household size					
One	41.87 (40.82, 42.92)	9.43 (8.76, 10.09)	12.73 (11.99, 13.47)	29.18 (28.29, 30.07)	6.78 (6.22, 7.36)
Two	39.82 (39.03, 40.61)	8.94 (8.45, 9.42)	14.16 (13.62, 14.70)	30.46 (29.73, 31.19)	6.62 (6.14, 7.11)
Three or more	37.00 (36.11, 37.88)	12.09 (11.49, 12.69)	20.54 (19.86, 21.23)	20.92 (20.21, 21.62)	9.45 (8.88, 10.02)

Note: Data shown as row percentage (95% confidence interval). The shown values are weighted percentages. ^a^ Divorced, widowed or separated. ^b^ Outside the center city of an MSA but inside the county containing the center city. ^c^ Inside a suburban county of the MSA.

**Table 2:** Odds ratios and 95% CIs from multinomial logistic regression of place of vaccination on individual characteristics, BRFSS 2018.

	Place of Vaccination (Ref Category: Hospital)			
Variables	Doctor’s Office	Health Department	Community Centers	Store
Chronic conditions				
0 (ref.)				
1–2	1.09 (0.88, 1.36)	0.93 (0.73, 1.20)	**0.66 (0.52, 0.84)**	0.92 (0.74, 1.14)
≥2	0.70 (0.42, 1.15)	0.81 (0.47, 1.42)	**0.35 (0.19, 0.63)**	**0.57 (0.34, 0.94)**
Smoking				
No (ref.)	**0.65 (0.47, 0.90)**	0.73 (0.51, 1.05)	0.72 (0.50, 1.04)	**0.69 (0.49, 0.96)**
Yes				
BMI category				
Normal	0.97 (0.77, 1.23)	0.94 (0.72, 1.22)	0.86 (0.67, 1.11)	1.14 (0.90, 1.45)
Above normal (ref.)				
Age category				
18–49 years (ref.)				
50–64 years	1.13 (0.79, 1.62)	0.91 (0.59, 1.42)	0.76 (0.51, 1.12)	0.92 (0.63, 1.33)
65+ years	**2.03 (1.42, 2.91)**	1.38 (0.88, 2.19)	**0.35 (0.22, 0.55)**	**1.85 (1.28, 2.68)**

Note: State-fixed effects were added to the regression model. Significant values in bold. The model included race, gender, marital status, health plan status, geographical location, income level, and household size.

**Table 3:** Odds ratios and 95% CIs from multinomial logistic regression of place of vaccination on COVID-19 risk category, BRFSS 2018.

	Place of Vaccination (Ref Category: Hospital)			
Variables	Doctor’s Office	Health Department	Community Centers	Store
COVID-19 risk				
Low risk (ref.)				
High risk	**1.52 (1.21, 1.91)**	**1.37 (1.06, 1.77)**	**0.54 (0.41, 0.70)**	**1.40 (1.12, 1.76)**

Note: State-fixed effects were added to the regression model. Significant values in bold. The model included race, gender, marital status, health plan status, geographical location, income level, and household size.

## Supplementary Materials

The following are available online at https://www.mdpi.com/article/10.3390/ijerph18157753/s1, Figure S1: Number of missing responses for each variable.
