# Supplementary material for: Using Influenza Vaccination Location Data from the 2018 Behavioral Risk Factor Surveillance System (BRFSS) to Expand COVID-19 Vaccination Coverage
Source: Int J Environ Res Public Health. 2021 Jul 22;18(15):7753. doi: 10.3390/ijerph18157753 (PMC8345551; doi:10.3390/ijerph18157753)
Supplement: Supplementary file 1 [file ijerph-18-07753-s001.zip › ijerph-1271787(XML suppl revised).pdf]

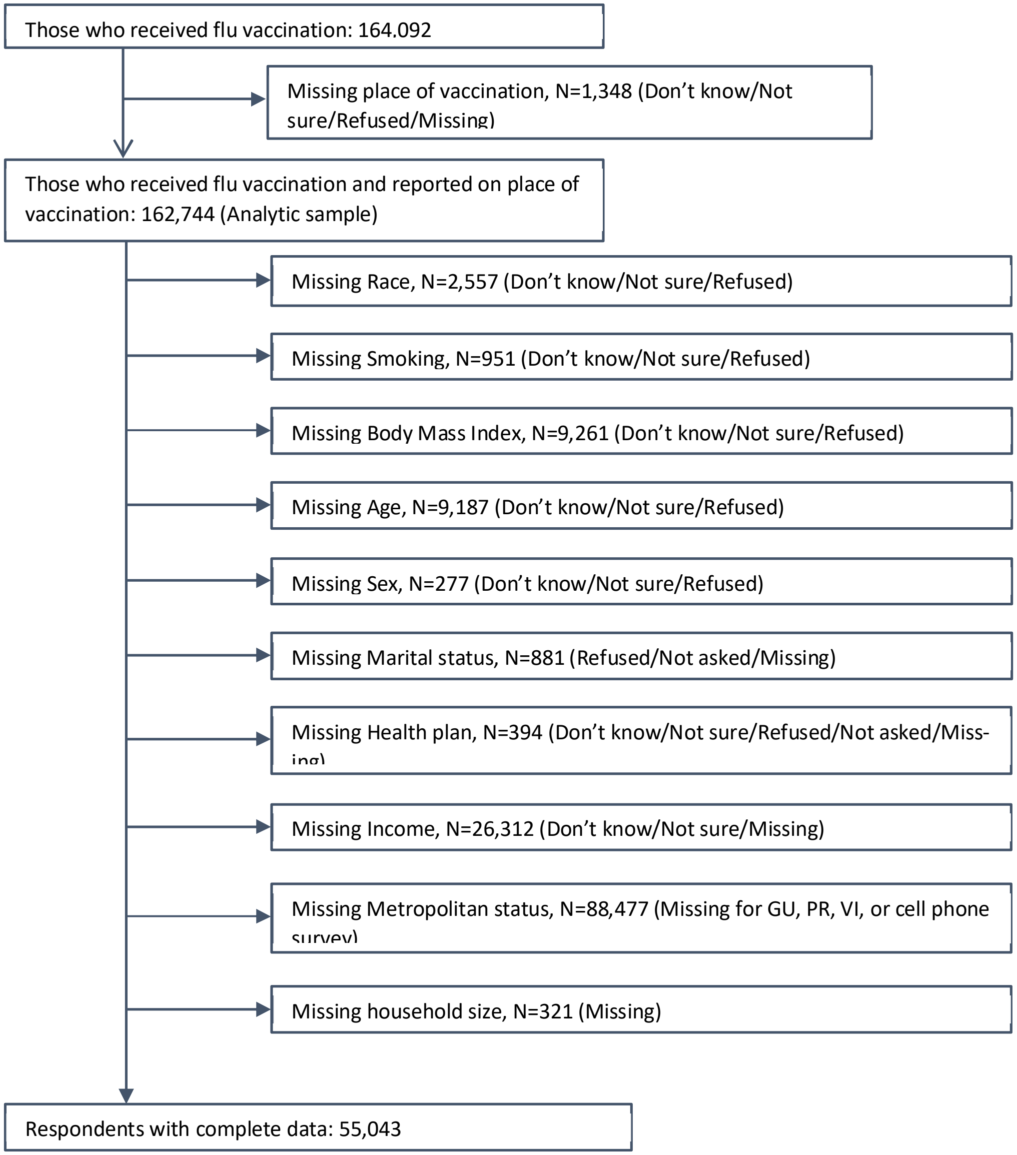

Figure S1. Number of missing responses for each variable.

Summary of significant covariates from the multinomial logistic regression of place of vaccination on individual risk factors

There were some significant differences based on sociodemographic characteristics (not shown in Table 2). Non-Hispanic black and white (vs. Hispanic) individuals had lower odds of vaccination at a health department (OR: 0.44, 95% CI: 0.22, 0.90 and OR: 0.48, 95% CI: 0.27, 0.88, respectively) and non-Hispanic black individuals had lower odds of vaccination at a store (OR: 0.47, 95% CI: 0.24, 0.92) than at a hospital. Those who were married (vs. never married/a member of an unmarried couple) had higher odds of receiving a vaccination at a doctor's office (OR: 1.44, 95% CI: 1.04, 1.99), community setting (OR: 1.78, 95% CI: 1.25, 2.52), or a store (OR: 1.56, 95% CI: 1.13, 2.17) than at a hospital. Those living in the center city of an MSA (vs. not in an MSA) had lower odds of receiving a vaccination at a health department (OR: 0.70, 95% CI: 0.51, 0.96) than at a hospital. However, those living in the MSA, either in the center city or county containing the center city (vs. those not living in the MSA), had higher odds of receiving a vaccination at a store (OR: 1.48, 95% CI: 1.14, 1.94; OR: 1.43, 95% CI: 1.04, 1.97 respectively) than at a hospital. Those with incomes <25,000 USD (vs. ≥25,000 USD) had lower odds of vaccination in community settings (OR: 0.40, 95% CI: 0.29, 0.55) than at hospitals.
